# Mucin-degrading gut bacteria: context-dependent roles in intestinal homeostasis and disease

**DOI:** 10.1080/19490976.2026.2614054

**Published:** 2026-01-23

**Authors:** Eunike Tiffany, Kyoung Su Kim, Panida Sittipo, Dong-Woo Lee, Yun Kyung Lee

**Affiliations:** a Department of Integrated Biomedical Science, Soonchunhyang Institute of Medi-Bio Science, Soonchunhyang University, Cheonan, Republic of Korea; b Department of Biotechnology, Yonsei University, Seoul, Republic of Korea; c Axe Cancer, Centre de recherche du Centre hospitalier de l’Université de Montréal (CRCHUM), Montréal, Québec, Canada; d Division of Pharmacology and Biopharmaceutical Sciences, Faculty of Pharmaceutical Sciences, Burapha University, Chonburi, Thailand

**Keywords:** Mucin-degrading bacteria, intestinal epithelial cells, immune cells, gut ecosystem

## Abstract

*Akkermansia muciniphila*, *Bacteroides thetaiotaomicron*, *Mediterraneibacter* (formerly *Ruminococcus*) *gnavus*, and other mucin-degrading (MD) bacteria play pivotal roles in shaping gut microbial ecosystems, maintaining gut barrier function, and mediating host–microbiota crosstalk. These bacteria influence intestinal homeostasis by modulating epithelial cell differentiation, immune responses, and gut microbiota composition through mucin degradation and the production of bioactive metabolites. Their abundance and functional activities fluctuate dynamically in response to dietary components, host immunity, and environmental factors, resulting in context-dependent effects on gastrointestinal and systemic health. This review summarizes current insights into the ecology and metabolic capabilities of MD bacteria, highlighting their dual roles in metabolic disorders, inflammatory diseases, infection susceptibility, and neuroimmune conditions. Understanding the ecological niches and molecular interactions of MD bacteria offers promising approaches for microbiota-targeted therapies aimed at restoring gut and systemic homeostasis.

## 1 Introduction

A healthy gut ecosystem is maintained through dynamic interactions between the gut microbiota and the host.^1^ The gut microbiota supports intestinal barrier function and shapes immune education through microbe-associated molecular patterns (MAMPs) and microbial metabolites.^2^^,^^3^ Disruption of this balance results in dysbiosis, which has been implicated in autoimmune and inflammatory disorders.^4^^-^^6^ In addition to host-microbiota crosstalk, stability of the gut ecosystem depends on microbial community interactions such as cross-feeding and mutualism,^7^^-^^9^ with each species contributing distinct metabolic capacities that enrich overall functionality.^10^

Within this complex community, only a small fraction of microbes—roughly 1% of the fecal microbiota—possess the unique ability to degrade mucin in the intestinal tract, particularly in the distal colon.^11^ These mucin-degrading (MD) bacteria employ a diverse repertoire of mucolytic enzymes, including glycoside hydrolases (GHs), sulfatases, sialidases, and peptidases to cleave glycans from the mucin backbone.^12^^,^^13^ Under homeostatic conditions, commensal MD activity is not harmful and is distinct from the MD strategies of pathogens.^14^ Importantly, their enzymatic repertoires differ substantially across species, conferring distinct ecological and metabolic profiles.^13^^,^^15^

Despite their relatively low abundance, MD bacteria occupy a critical ecological niche.^16^ Prominent representatives include *Akkermansia muciniphila*, *Mediterraneibacter* (formerly *Ruminococcus*) *gnavus*, *Bacteroides* spp., *Bifidobacterium* spp., and *Barnesiella intestinihominis,* many of which function synergistically in the mucosal environment.^14^^,^^15^ Among these, *A. muciniphila* is the most extensively studied and is often proposed as a next-generation probiotic.^17^^,^^18^ Its abundance has been linked to improved microbial community structure,^19^ epithelial and immune activation,^20^^,^^21^ and enhanced responses to immune checkpoint inhibitors.^22^ Paradoxically, elevated levels of *A. muciniphila* have also been associated with autoimmune diseases^23^^,^^24^ and inflammatory pathologies.^25^^,^^26^ Similarly, although less studied, other MD bacteria also demonstrate dual roles in disease. For instance, *M. gnavus* is frequently enriched in gastrointestinal autoimmune disorders^27^^-^^29^ but has also shown protective effects in certain contexts.^30^^-^^32^ These contrasting findings highlight the context-dependent role of MD bacteria in health and disease. Emerging evidence further points to strain-level variation and differential metabolic activities as important determinants of these divergent outcomes.^33^^-^^36^ Yet, many MD bacteria remain poorly characterized, leaving their ecological roles, host interactions, and defining features as keystone species largely unexplored.^12^

In this review, we summarize and integrate current knowledge of the ecological drivers, host interactions, and disease-related roles of MD bacteria. We highlight the factors influencing their abundance and phenotypes, their contributions to intestinal barrier and immune regulation, and the mechanisms through which they exert both protective and pathogenic effects. Understanding these dualistic roles will be essential for harnessing MD bacteria in microbiota-targeted therapies. In the following sections, we first examine the ecological and environmental drivers of MD bacteria, then their bidirectional interactions with the host, and finally their context-dependent roles in health and disease.

## 2 Ecological and environmental drivers of MD bacteria

Under normal conditions, MD bacteria maintain basal MD activity while signaling goblet cells to secrete mucin.^37^^,^^38^ They preferentially utilize free glycans, and in doing so, release cleaved glycans that become accessible to other microbes, thereby shaping microbial community structure and contributing to mucus turnover.^37^^,^^39-41^ However, disturbances such as altered mucosal barrier function or dietary changes can shift the abundance and activity of MD bacteria, with downstream effects on the gut ecosystem. In this section, we focus on how nutrient availability and cross-feeding interactions regulate MD bacterial behavior.

### 2.1 Nutrient-dependent regulation of MD bacterial abundance and activity

Most gut microbes rely on indigestible dietary nutrients such as fiber for carbon and unabsorbed amino acids for nitrogen.^42^^,^^43^ Notably, shifts in dietary composition can induce microbial dysbiosis and modulate the MD bacterial activity.^44^ Fiber-rich diets support fiber-degrading microbes via *α*- and *β*-glucanases.^45^ For instance, *β*-glucan-rich fiber promotes the growth of beneficial gut microbes such as *Bifidobacterium* and *Lactobacillus,*^46^ whereas soluble fiber (e.g., inulin, pectin, arabinoxylans, resistant dextrins, etc.) promote beneficial taxa, including *Bifidobacterium* spp. and butyrate producers like *Faecalibacterium prausnitzii.*^47^ In contrast, fiber-free diets force MD bacteria to utilize host mucin as a nutrient source, leading to degradation of the protective mucus barrier.^48^^,^^49^

In fiber-free conditions, *A. muciniphila* and *B. caccae* increase, while *Barnesiella intestinihominis* and *B. thetaiotaomicron* decrease.^48^ Such conditions often elevate MD activity in a subset of MD bacteria and promote pathobiont-like behavior that compromises barrier integrity.^49^ Exclusive enteral nutrition similarly promotes *M. gnavus* expansion and disrupts MD enzyme expression.^50^ High-fat diet (HFD) impairs goblet cell mucus secretion and can reduce *A. muciniphila* abundance by up to 100-fold.^33^^,^^51^^,^^52^ Akkermansiaceae and Bacteroidaceae also decrease under HFD but are restored with inulin supplementation, highlighting the importance of fiber for balanced MD activity.^33^ Western-style diets also shift the genomic profile of *B. thetaiotaomicron*, enhancing mucin O-glycan utilization and increasing *A. muciniphila* abundance.^53^ Thus, nutrient shifts alter both population dynamics and enzymatic activity.^33^^,^^34^

*A. muciniphila* thrives on mucin, upregulating MD enzymes and producing metabolites such as propionate and indole derivatives under mucin-rich conditions.^33^^,^^40^ In contrast, *M. gnavus*, with fewer MD enzymes, grows poorly on intact mucin but efficiently utilizes mucin-derived sugars (e.g., fucose, galactose), producing metabolites such as propionate and indole derivatives.^13^^,^^33^ *M. gnavus* can also metabolize 2,7-anhydro-Neu5Ac (a form of sialic acid) via an intramolecular trans-sialidase (IT-sialidase) encoded within the Nan cluster,^13^ altering its metabolic outputs by reducing fumarate and citrulline production.^54^

Human milk oligosaccharides (HMOs), structurally similar to mucin glycans, enrich *A. muciniphila* and *B. caccae* while reducing *M. gnavus.*^55^^,^^56^ Specific HMOs (e.g., 2-fucosyllactose) increase cecal propionate, reduce colonic inflammation in IL10^⁻/⁻^ mice, and modulate MD activity of *M. gnavus.*^31^ HMO supplementation also increases *A. muciniphila* abundance and short-chain fatty acid (SCFA) production in healthy mice^57^ and improves metabolic outcomes such as obesity through enhanced mucin secretion and glycosylation.^58^ Protein content also influences MD populations^59^; high-casein diets enhance *B. thetaiotaomicron* and sialidase activity, thinning the mucus barrier.^60^ Certain amino acids are essential for growth—e.g., *A. muciniphila* requires threonine,^40^^,^^61^ while serine-depleted diets promote its expansion in DSS models, enhancing pathogenic mucin degradation.^62^

### 2.2 Microbial interactions and cross-feeding networks

MD bacteria shape gut ecology not only by consuming host-derived glycans but also by releasing monosaccharides and fermentation products that serve as shared substrates for other commensal bacteria. These cross-feeding interactions reshape microbial composition and reinforce cooperative microbial network.^39^

In murine models of inflammation or immunosuppression, *A. muciniphila* promotes the expansion of beneficial taxa such as Muribaculaceae, *Lachnoclostridium*, and *Parabacteroides goldsteinii*, while reducing pathogens including Gammaproteobacteria, *Escherichia-Shigella*, and Enterobacteriaceae.^19^ In inflammatory bowel disease (IBD) mouse models, *A. muciniphila* enhances microbial diversity by enriching Bacillota, Ruminococcaceae, and *Oscillibacter*, while suppressing *Bacteroides.*^63^ Stable colonization further altered host metabolic pathways, particularly those related to carbon and amino acid metabolism.^8^

Like *A. muciniphila*, other MD bacteria act as keystone species contributing to the expansion and diversity of beneficial gut taxa. *Bifidobacterium bifidum* expresses sulfoglycosidases that releases *N*-acetylglucosamine-6-sulfate, potentially modulating broader community metabolism.^39^ In addition, *Bacteroides fragilis* and *Bacteroides stercosis* function as key carbohydrate degraders producing SCFAs and secondary bile acids,^64^ which support the growth of butyrate producers such as *F. prausnitzii*.

MD bacteria also directly influence the metabolic activity of other microbes. *A. muciniphila* produces fucosidases (GH29/95) and sialidases (GH33) that liberate sugars supporting the growth of butyrate-producing *Clostridia.*^65^ Its fermentation products, including SCFAs, can serve as substrates for *Anaerobutyricum hallii*, which in turn produces pseudovitamin B₁₂.^7^ Similarly, *B. thetaiotaomicron* facilitates the colonization of *F. prausnitzii* by generating acetate, a precursor for additional SCFA production.^37^ When stimulated by dietary pectin, *B. thetaiotaomicron* degrades polysaccharides into monosaccharides while simultaneously suppressing microbial indole production,^66^ highlighting its role as a mucin “generalist” with broad ecological influence.

Genomic analyses confirm functional diversity among MD bacteria.^67^ *A. muciniphila* and *B. thetaiotamicron* share GHs such as GH16, GH109, and carbohydrate-binding modules (CBM32), reflecting their capacity to initiate mucin degradation.^33^^,^^68^^,^^69^ By contrast, *M. gnavus* carries a distinct repertoire (e.g., GH98 and CBM40), positioning it as a metabolic “follower”.^33^^,^^70^ Rather than initiating mucin breakdown, *M. gnavus* competes with *M. bromii* for glucose and malto-oligosaccharides, prompting *M. bromii* to upregulate tryptophan- and vitamin B₁₂-dependent methionine biosynthesis pathways.^70^

Together, these interactions illustrate how MD bacteria not only secure their own niche but also orchestrate broader community metabolism through cross-feeding. In the next section, we shift from ecological networks to examine how MD bacteria interact directly with the host epithelium and immune system.

## 3 Interplay between MD bacteria and the host

Intestinal epithelial cells (IECs) serve as both a physical and immunological barrier between the host and the gut lumen. Under homeostatic conditions, MD bacteria are primarily confined to the dynamic outer mucus layer.^14^ However, under pathological or environmental stress, the inner mucus layer can become permeable, enabling direct contact between MD bacteria, IECs, and mucosal immune cells.^4^^,^^71^ These interactions are highly context-dependent, influenced by bacterial species, enzymatic repertoires, and host immune status. Here, we examine how MD bacteria both shape and respond to host epithelial and immune function (Table 1).

**Table 1.** The effect of MD bacteria and its derivatives on host IEC and immune cells.

MD bacteria		Metabolites/bacterial component	Effect on host cells	Mechanism of action	Experiment set up	Ref
*Akkermansia muciniphila*	Epithelial cells	SCFA	Increase mucus production			[40,52]
		Acetate, propionate	Increase Lgr5 ^+^ stem cell proliferation, Paneth cell and goblet cell differentiation	GPR41, GPR43	SPF C57BL/6 mice, small intestinal organoid	[38]
		Amuc_1409	Increase intestinal stem cell proliferation	Wnt/β-catenin signaling	Mouse small intestinal organoid, total body gamma irradiation	[72]
		P9	GLP-1 secretion by L cells	ICAM2	High fat diet-fed mice, NCI-H716 cells	[73]
		Amuc_1100	Epithelial regeneration	TLR2-TRAF6, CREB	DSS-induced mice, Caco-2 cell, IPEC-J2 cell	[74]
		Propionate	Enhance tight junctions protein	Gpr41 Gpr43	IL-1β induced Caco-2 cells	[75]
		Amuc_2109	Enhance tight junctions protein		DSS-induced mice	[76]
	Immune cells	Amuc_1100	NF-κβ activation	TLR2	PBMC	[77,78]
		a15:0-i15:0 PE	TNFα	TLR1/TLR2	BMDC	[79]
		Ornithine lipid	Reduce LPS-induced inflammation	Atf3	LPS-induced BMDM	[80]
		Threonyl-tRNA synthetase	IL-10	TLR2	Naïve and LPS-induced BMDM, DSS-induced colitis	[81]
			Retinoic acid	JAK2-STAT3, RALDH2	DSS-induced colitis, CD103 ^+^ DCs	[82]
		EVs	Clade-specific IgA		Germ-free mice	[83]
			Antigen-specific IgG1	PD-1 expressing T follicular helper	Gnotobiotic mice	[20]
*Mediterraneibacter gnavus*	Epithelial cells	Small peptide	Enhance Muc2 expression, mucus glycosylation	ST6GAL1, MGAT3, B4GALT1, B3GNT6	Germ-free mice, HT29-MTX cell	[84]
		Tryptamine, phenetylamine	Increase serotonin production by enterochromaffin cells	TAAR1	Germ-free mice, spontaneous diabetes monkey, 3T3-L1 cell	[27]
			Reg3g, Reg3b, and lysozyme-1 expression in Paneth cells	MyD88	SPF C57BL/6 mice	[85]
			IL-25 secretion of Tuft cells		Antibiotic-treated Lyz^-/-^ mice	[86]
		Arginine metabolites	Nitric oxide	NOS2	Germ-free mice	[54]
	Immune cells	Glucorhamnan I	TNFα, IL-6, IL-12p40	TLR4, NF-κβ	BMDC	[29]
		Glucorhamnan II	NF-κβ activation	TLR4	BMDC	[87]
		Absence of capsular polysaccharide	TNFα		BMDC, germ-free mice	[88]
*Bacteroides thetaiotaomicron*	Epithelial cells	Acetate	Increase mucus production		Germ-free rat, HT29-MTX cell	[37]
			Enhance mucus fucosylation, increase mucus sialylation-to-sulfation ratio		Germ-free mice	[37]
			Enhance ZO-1, Claudin-1, E-cadherin		IL-1β induced Caco-2 cells	[75]
	Immune cells	EVs	IL-10, IL-6, CD80	NF-κβ activation	PBMC-derived dendritic cells	[89]
*Bacteroides fragilis*	Immune cells	PSA	IL-10	TLR2	Splenic DCs, PBMC derived plasmacytoid DC, *H. hepaticus*-induced colitis	[90,91]
		Sphingolipid	iNKT cell in colonic lamina propria	CD1d	Mono-colonized germ free mice	[92]
*Bacteroides acidofaciens*	Immune cells		IgA	Activation-induced cytidine deaminase	Mono-colonized germ free mice	[93]
*Bacteroides salyersae*	Epithelial cells		Enhance Claudin-1 and E-cadherin		Caco-2 monolayer	[75]
*Parabacteroides distasonis*			Enhance Occludin		IL-1β-induced Caco-2 cells	[75]
*Barnesiella intestinihominis*	Epithelial cells		Enchance ZO-1 and Occludin		Caco-2 monolayer	[94]
	Immune cells		Ath1 and Tc1 induction		Cyclophosphamide-treated tumor bearing mice	[95]

### 3.1 Epithelial regulation and barrier integrity

The structural integrity and secretory activity of IECs are central in regulating MD bacterial dynamics. Disruption of epithelial function—through genetic mutations, altered glycosylation, or epithelial damage—reshapes the mucosal environment and influences MD bacterial abundance.

In *Muc2*-deficient mice, which lack the major mucus component, *Bacteroides* spp. and *A. muciniphila* dominate, whereas other MD bacteria such as Ruminococcaceae are depleted. This shift results dysbiosis and altered CNS-related behavior through systemic metabolic remodeling.^96^^,^^97^ This *Akkermansia*-dominated community is characterized by reduced microbial diversity and fewer butyrate-producing commensals, forming a disease-locked microbial state associated with mucus barrier defects. Similarly, Winnie mice, which carry a missense *Muc2* mutation and display a less compact mucus layer, show altered MD bacteria composition due to increased access to simple mucin sugars, including higher *A. muciniphila* and *B. acidifaciens* and lower *Barnesiella intestinihominis.*^98^

By contrast, loss of FoxO1, a regulator of mucin secretion, decreases *A. muciniphila* while increasing *M. gnavus* and *M. torques* and elevating mucinase activity,^99^ resulting in impaired barrier integrity, increased inflammation, and systemic immune-metabolic dysfunction. *Gpr35* knockout impairs goblet cell differentiation, leading to dysbiosis with *A. muciniphila* depletion, *M. gnavus* and *Bacteroides* expansion, and altered aromatic amino acid metabolism which promotes HFD-induced liver steatosis.^100^

Glycosylation also dictates MD bacterial colonization. *Fut2* mutations, which impair mucosal fucosylation, reduce *A. muciniphila,*^101^ whereas *Fut2* upregulation driven by Na⁺/H⁺ exchanger 3 promotes *B. thetaiotomicron* growth.^102^ Loss of *ST6GAL1*-mediated α2,6-sialylation lowers *Mediterraneibacter* while modestly increasing *Bacteroides.*^103^ Depletion of group 3 innate lymphoid cells (ILC3s) enhances mucosal *β*-1,3-galactosylation, promoting *A. muciniphila* expansion and succinate production, which drives pathogen virulence and exacerbates inflammation.^104^ Enteroendocrine cell deficiency decreases *Akkermansia* while increasing *Bacteroides* and *Bifidobacterium.*^105^ Lysozyme deficiency elevates goblet and tuft cell numbers, driving *M. gnavus* expansion and even inducing anti-inflammatory responses not observed in wild-type mice.^106^

**Figure 1.**
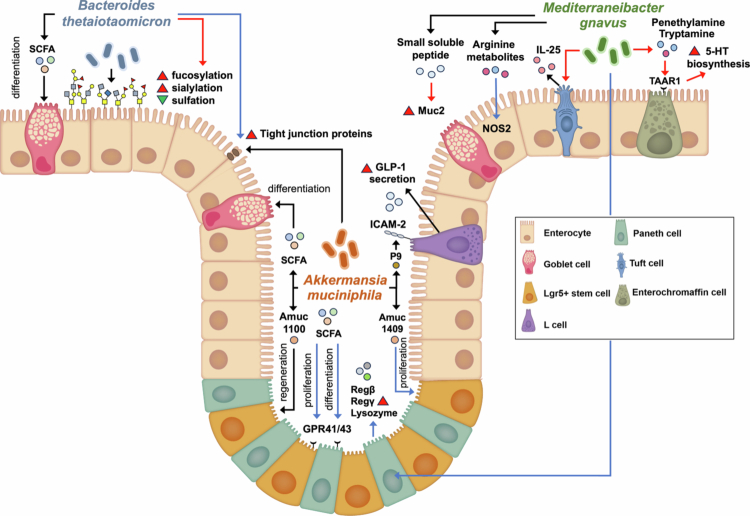
Overview of the mechanisms by which MD bacteria, including *A. muciniphila*, *B. thetaiotaomicron*, and *M. gnavus*, transmit signals to IECs, thereby enhancing the differentiation, proliferation, and functions of specific IEC lineages through microbial component and metabolites. Black arrow refers to events happening in both colon and small intestine, red arrow refers to events happening in colon only, while blue arrow refers to events happening in small intestine only.

In proximity to IECs, MD bacteria modulate epithelial function through metabolites and secreted proteins. SCFAs generated during mucin metabolism, particularly acetate and propionate, provide energy for goblet cell mucin synthesis.^37^^,^^107^ MD bacteria with mucus-binding capability enhance barrier integrity by inducing filamentous mucin secretion from intercrypt goblet cells.^108^ *A. muciniphila* promotes proliferation of Lgr5⁺ intestinal stem cells and differentiation of Paneth and goblet cells in the small intestine through SCFAs signaling via G protein-coupled receptors (GPR)41 and GPR43.^38^ It also secretes bioactive proteins such as Amuc_1409, which activates Wnt/β-catenin signaling to stimulate stem cell proliferation.^72^ P9, an 84-kDa protein, binds intercellular adhesion molecule-2 on L-cells to induce glucagon-like peptide-1 secretion,^73^ while the surface protein Amuc_1100 enhances epithelial regeneration by activating TLR2–TRAF6 and CREB signaling and suppressing endoplasmic reticulum stress via insulin-like growth factor pathways^74^ Figure 1.

*M. gnavus* modulates IECs through multiple mechanisms. It alters mucus glycosylation patterns and enhances *Muc2* expression in goblet cells via small peptide production.^84^ It also activates colonic trace amine-associated receptor 1 in enterochromaffin cells through phenethylamine and tryptamine production—implicated in the pathophysiology of irritable bowel syndrome (IBS)^27^—and induces Paneth cell antimicrobial responses (*Reg3g*, *Reg3b*, and *lysozyme-1*) via MyD88-dependent signaling.^85^ *M. gnavus* increases tuft cells and IL-25 secretion in the proximal colon, amplifying type 2 immune responses.^86^ It also induces nitric oxide synthase 2 (NOS2) expression in the small intestine via arginine derivatives, strengthening the barrier, although this pathway is suppressed in the presence of sialic acid, a condition common in intestinal inflamation.^54^

Multiple *Bacteroides* and *Parabacteroides* strains enhance barrier integrity by upregulating tight junction genes in Caco-2 cells. *B. thetaiotaomicron* increases the expression of ZO-1, Claudin-1, and E-cadherin, while *P. distaonis* specifically upregulates Occludin. *B. salyersiae* increased Claudin-1 and E-cadherin. These strains also partially restore TEER during IL-1β-challenge.^75^ *B. thetaiotaomicron* produces acetate and propionate—acetate specifically promotes KLF4 expression in mucus-producing HT29-MTX cells—and enhances colonic mucus fucosylation, altering sialylation-to-sulfation ratios in germ-free mice.^37^

Together, these findings highlight a reciprocal relationship: epithelial genetic and cellular contexts dictate MD bacterial colonization, while MD species, in turn, regulate epithelial physiology. In the next section, we address how MD bacteria are shaped by the immune system and actively modulate innate and adaptive immune responses, reinforcing their context-dependent roles in health and disease.

### 3.2 Immunomodulatory roles of MD bacteria

Like other members of the gut microbiota, MD bacteria communicate with host immune cells through MAMPs, secreted proteins, and metabolites (Table 1). These signals are detected by PRRs and shape both innate and adaptive immune responses.

**Figure 2.**
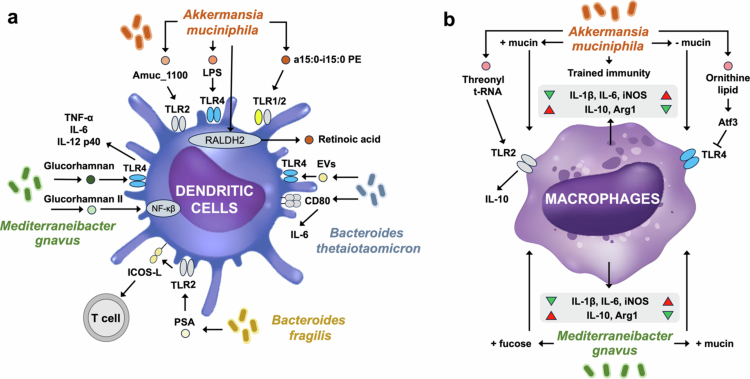
Summary of the interactions between MD bacteria (e.g., *A. muciniphila*, *B. thetaiotaomicron*, *M. gnavus*, and *B. fragilis*) and the innate immune systems. These bacteria activate PRRs, such as TLR2, TLR4, and TLR1/2 on immune cells, modulating their activation status and cytokine production. (a) The effect of MD bacteria on dendritic cells. (b) The role of MD bacteria on macrophages.

**Figure 3.**
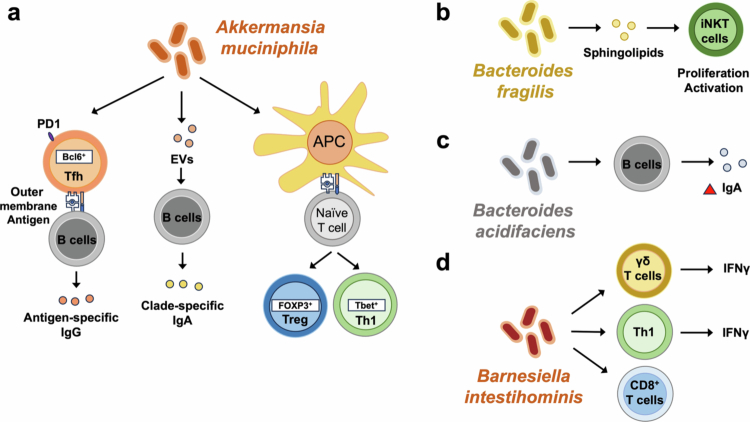
Roles of MD bacteria in the differentiation and function of adaptive immune cells. (a) *A. muciniphila*, (b) *B. fragilis*, (c) *B. intestinihominis*, and (d) *B. acidifaciens* role.

#### 3.2.1 Innate immune modulation

*A. muciniphila* engages multiple innate signaling pathways. Its membrane-associated macromolecules, including lipopolysaccharide (LPS), Amuc_1100,^77^^,^^78^ and diacyl phosphatidylethanolamine (a15:0-i15:0)^79^ activate different TLR signaling cascades and induce pro-inflammatory responses in immune cells (Figure 2). Additional effects include enhanced retinoic acid dehydrogenase expression in DCs via JAK2–STAT3 signaling, leading to increased IL-22 production by ILC3s.^82^ Depending on nutrient context, *A. muciniphila* can reduce pro-inflammatory and enhance anti-inflammatory cytokines in macrophages grown in mucin-rich media.^33^ It also induces trained immunity in macrophages, strengthening phagocytic and bactericidal functions while limiting cytokine hyperactivation.^109^ Secreted threonyl-tRNA synthetase activates TLR2–CREB signaling to promote M2 macrophage polarization and IL-10 production,^81^ while ornithine lipids upregulate the anti-inflammatory gene *Atf3*, a TLR4 inhibitor with anti-inflammatory properties.^80^

*M. gnavus* exhibits strain- and nutrient-dependent immunogenicity. Certain strains possess a capsular polysaccharide that masks immune recognition, minimizing TNF-*α* induction in BMDCs.^88^ In contrast, strain ATCC 29149 produces glucorhamnan-I, an exopolysaccharide that activates TLR4-NF-κβ signaling and induces TNF-*α*, IL-6, and IL-12p40.^29^ Other strains (e.g., E1, ATCC 35913) produce glucorhamnan-II with distinct branching, eliciting stronger NF-κβ activation.^87^ Nutrient conditions further shape outcomes: growth in mucin promotes pro-inflammatory cytokines (IL-6, IL-1β), whereas fucose supplementation induces IL-10.^33^

*B. thetaiotaomicron* secretes extracellular vesicles (EVs) that cross the mucus barrier and activate macrophages and DCs via TLR4–NF-κB.^110^ These EVs modulate IL-10 production in colonic DCs from healthy donors but not IBD patients, indicating host-dependent responses.^89^ They also enhance IL-6 and CD80 expression in peripheral DCs.^89^ *B. fragilis* produces polysaccharide A (PSA), which stimulates TLR2 on plasmacytoid DCs, promoting co-stimulatory molecules and IL-10-producing T cells.^90^

#### 3.2.2 Adaptive immune modulation

The absence of mature T and B cells increases *A. muciniphila* abundance, reaching up to 10% of the total gut microbiota.^111^ Moreover, impaired IgA-mediated bacterial containment due to disrupted IL-33 signaling results in expansion of *A. muciniphila* and elevated MD activity, suggesting that the adaptive immune system normally restricts the overgrowth of this bacterium.^112^ Conversely, peripheral Tregs (pTregs) support *A. muciniphila* colonization; in mice lacking pTregs, this bacterium is nearly undetectable.^113^

In turn, *A. muciniphila* also shapes adaptive immunity. Supplementation of *A. muciniphila* restores epithelial and immune balance by inducing RORγt⁺ regulatory T (Treg) cells in TLR4-deficient mice, in which *A. muciniphila* was depleted.^21^ It promotes antigen-specific T cell responses and IgG1 production in a T follicular helper (Tfh)-dependent manner,^20^ increases PD-1 and Bcl6 in Tfh cells, and drives clade-specific IgA production by B cells, partly mediated through EVs^83^ (Figure 3). It enhances both thymic and peripheral Tregs differentiation^113^ and can promote anergy or Treg differentiation from naïve CD4⁺ T cells.^114^ However, in other contexts (e.g., multiple sclerosis (MS)), it can skew PBMCs toward Th1 responses with increased IFN-*γ* and T-bet expression.^115^

Other MD bacteria also exert adaptive immune effects (Figure 3). Restoration of MD bacteria by γδ T cell-derived let-7f microRNA and *M. gnavus* administration rescues oral tolerance defects in γδ T cell-deficient mice.^116^ *M. gnavus* also supports anti-tumor CD8⁺ T cell activation through degradation of lysoglycerophospholipids.^32^ *B. fragilis*-derived sphingolipids suppress invariant natural killer T cell proliferation and function in colonic lamina propria, exacerbating colitis.^92^ *Barnesiella intestinihominis* enhances antitumor immunity by recruiting IFN-γ⁺ γδ T cells, Th1 cells, and cytotoxic CD8⁺ T cells.^95^ *Bacteroides acidifaciens* monocolonization increases colonic IgA production and IgA⁺ B cell populations, reinforcing barrier immunity.^93^

Collectively, these studies highlight that immune system not only alters the abundance of MD bacteria but can also shift their behavior toward pathobiont-like phenotypes. Furthermore, these findings emphasize the dualistic immunomodulatory capacity of MD bacteria, which can promote either tolerance or inflammation depending on microbial strain, nutrient context, and host immune status. This duality underscores their potential as both biomarkers and therapeutic targets, setting the stage for understanding their context-dependent roles in gut and systemic diseases.

## 4 Context-dependent roles of MD bacteria in disease

Given their intimate involvement in epithelial and immune regulation, MD bacteria display strikingly context-dependent roles in health and disease. Their abundance and activity fluctuate with diet, host genetics, and microbial ecology, which in turn shape their capacity to protect or disrupt gut homeostasis. Furthermore, alterations in the abundance of MD bacteria have been observed in clinical studies, and some have even been proposed as potential disease biomarkers (Table 2). In this section, we summarize their dualistic contributions across gastrointestinal and extra-intestinal disorders and highlight the determinants that govern these divergent outcomes.

**Table 2.** MD bacteria and its abundance alteration in clinical studies.

Genus	Species	Strain	Acession number	Association with diseases	Ref
Akkermansia	*Akkermansia muciniphila*	ATCC BAA-835	NR_074436.1	Higher in: Multiple sclerosis, rheumatoid arthritis, colorectal cancer	[117-120]
				Lower in: Ulcerative colitis, obesity, atopic dermatitis, psoriasis	[121-125]
				Unconclusive: Alzheimer disease	[126,127]
*Mediterraneibacter*	*Mediterraneibacter gnavus*	ATCC 29149	NR_036800.1	Higher in: Chron's disease, ulcerative colitis, multiple sclerosis, Alzheimer's disease, lupus nephritis, psoriasis, allergy	[33,122,124,125,128-132]
				Lower in: Atopic dermatitis	[133]
				Unconclusive:	
	*Mediterraneibacter torques*	ATCC 27756	L76604.1	Higher in: Chron's disease, ulcerative colitis, multiple sclerosis, obesity	[123,125,128,132]
				Lower in:	
				Unconclusive:	
Bificobacterium	*Bifidobacterium bifidum*	ATCC 35914	AB437356.1	Higher in: Parkinson's disease, Chron's disease	[11,128,134]
				Lower in: Allergy	[129]
				Unconclusive:	
	*Bi. longum subsp. longum*	NCIMB 8809	CP011964.1	Higher in: Parkinson's disease	[134,135]
				Lower in: Ulcerative colitis, Chron's disease, obesity, allergy	[121,123,129]
				Unconclusive:	
	*Bi. longum subsp. infantis*	ATCC 15697	M58738.1	Higher in:	[136]
				Lower in:	
				Unconclusive:	
	*Bi. breve*	UCC2003 (NCIMB 8807)	CP000303.1	Higher in:	[137]
				Lower in: Ulcerative colitis, obesity	[121,138]
				Unconclusive:	
Bacteroides	*Bacteroides caccae*	ATCC 43185	AB510697.1	Higher in:	[139]
				Lower in: Chron's disease	[128]
				Unconclusive:	
	*B. dorei*	DSM 17855	AB242142.1	Higher in:	[139]
				Lower in: Allergy	[129]
				Unconclusive:	
	*B. fluxus*	DSM 22534	AB547642.1	Higher in:	[139]
				Lower in:	
				Unconclusive:	
	*B. fragilis*	NCTC 9343, ATCC 25285	AB510701.1	Higher in:	[139]
				Lower in:	
				Unconclusive: Chron's disease, ulcerative colitis	[128]
	*B. massiliensis*	B84634, DSM 17679	AB510703.1	Higher in:	[139]
				Lower in:	
				Unconclusive:	
	*B. nordii*	CL02T12C05	NZ_JH724315.1	Higher in:	[139]
				Lower in:	
				Unconclusive:	
	*B. ovatus*	NLAE-zl-H361	AB510705.1	Higher in: Chron's disease	[128,139]
				Lower in: Allergy	[129]
				Unconclusive: Atopic dermatitis	[140]
	*B. plebeius*	DSM 17135	AB200217.1	Higher in:	[139]
				Lower in:	
				Unconclusive:	
	*B. salyersae*	VPI-2828	LR999593.1	Higher in:	[139]
				Lower in:	
				Unconclusive:	
	*B. thetaiotaomicron*	VPI-5482, ATCC 29148	M58763.2	Higher in:	[139]
				Lower in: Atopic dermatitis	[140]
				Unconclusive: Chron's disease, ulcerative colitis	[128]
	*B. vulgatus*	ATCC 8482	AB510712.1	Higher in: Chron's disease, ulcerative colitis	[128,139]
				Lower in:	
				Unconclusive:	
	*B. xylanisolvens*	NLAE-zl-H194	AB510713.1	Higher in:	[139]
				Lower in: Atopic dermatitis	[140]
				Unconclusive:	
Parabacteroides	*Parabacteroides distasonis*	ATCC 8503	NR_041342.1	Higher in: Parkinson's disease	[134,139]
				Lower in: Alzheimer's disease, psoriasis	[130,131]
				Unconclusive:	
	*P. goldsteinii*	DSM 19448, WAL 12034	AB547650.1	Higher in:	[139]
				Lower in:	
				Unconclusive:	
	*P. gordonii*	DSM 23371	AB470343.1	Higher in:	[139]
				Lower in:	
				Unconclusive:	
	*P. johnsonii*	DSM 18315	AB261128.1	Higher in:	[139]
				Lower in:	
				Unconclusive:	
	*P. merdae*	ATCC 43184	AB238928.1	Higher in:	[139]
				Lower in: Atopic dermatitis	[140]
				Unconclusive:	
Barnesiella	*Barnesiella intestinihominis*	DSM 21032	AB370251.1	Higher in:	[139]
				Lower in: impaired glucose regulation, type 2 diabetes mellitus, obesity, Chron's disease	[94,123,128]
				Unconclusive:	
Clostridium	*Clostridium celatum*	WC 0700	NZ_JAJKGM010000097.1	Higher in:	[141]
				Lower in:	
				Unconclusive:	
	*C. tertium*	WC 0709	NZ_JAHLZG010000025.1	Higher in:	[141]
				Lower in:	
				Unconclusive:	
Paraclostridium	*Paraclostridium bifermentans*	WC 0705	NZ_JAJLQV010000035.1	Higher in:	[141]
				Lower in:	
				Unconclusive:	
Anaerotruncus	*Anaerotruncus colihominis*	DSM 17241	AJ315980.1	Higher in:	[142]
				Lower in:	
				Unconclusive:	
Marvinbryantia	*Marvinbryantia formatexigens*	DSM 14469	NR_042152.1	Higher in:	[142]
				Lower in:	
				Unconclusive:	
Peptostreptococcus	*Peptostreptococcus russellii*	DSM 23041	AY167952.1	Higher in:	[142]
				Lower in:	
				Unconclusive:	

### 4.1 Gastrointestinal disorders

The dualistic nature of MD bacteria is most evident in gastrointestinal diseases, including inflammatory bowel disease (IBD), IBS, and colorectal cancer (CRC).^42^ Their abundance and activity fluctuate across disease states, reflecting both protective and pathogenic capacities.

#### 4.1.1 Inflammatory bowel disease (IBD)

In ulcerative colitis (UC) and Crohn’s disease (CD), mucosal barrier disruption is accompanied by reduced mucin peptide and altered glycosylation.^143^ Changes in the intestinal mucus layer drive an MD bacterial signature characterized by reduced fucosidase capacity and induction of glycan transporter systems.^142^ At the species level, patients with UC commonly exhibit diminished *A. muciniphila* and *Bifidobacterium* spp., along with increased *M. gnavus* and *Bacteroides* spp.^121^^,^^128^^,^^144^^,^^145^. In CD, *M. gnavus* abundance is consistently elevated, whereas *A. muciniphila* levels tends to decrease in some reports.^128^^,^^144^

A particular *M. gnavus* clade increases transiently in IBD patients, displaying distinct genotypes related to oxidative stress response, mobile elements, and mucus utilization.^28^^,^^146^ This transient bloom may be driven by its adaptability to the highly sialylated mucin observed in IBD. *M. gnavus* strains harboring the Nan-cluster can outcompete other bacteria in this niche and produce fewer beneficial metabolites important for host defense.^54^^,^^143^^,^^147^ *Bacteroides* spp. also increase transiently in UC and CD, although their competitive mechanisms remain unclear.^128^

Mouse models underscore the strain- and environment-dependent roles of *A. muciniphila*. Overall, this bacterium shows protective capacity in IBD by reducing pro-inflammatory cytokines and enhancing barrier function.^121^^,^^148^^,^^149^ Stable colonization supports its beneficial effects through NLRP3 inflammasome activation, strengthening barrier integrity and reducing systemic inflammation.^63^^,^^121^ Conversely, *A. muciniphila* overexpansion under serine-depleted diets^62^ or in fiber-free fed IL10^-/-^ mice induces excessive mucin degradation and barrier disruption, thereby exacerbating colitis.^71^ It has also been implicated in CRC progression by impairing mucosal integrity, increasing IEC proliferation, and elevating pro-inflammatory cytokines.^26^^,^^117^ However, its outer membrane protein Amuc_1100 can activate PD-1^+^ CD8^+^ cytotoxic T lymphocytes and inhibit tumorigenesis.^150^

*M. gnavus* also exhibits context-dependent behavior. In germ-free mice, monocolonization increases phenethylamine and tryptamine production, stimulating enterochromaffin cell serotonin release and contributing to diarrhea-predominant IBS.^27^ In IL10^-/-^ mice, *M. gnavus* blooms in the presence of 2-fucosyllactose (an HMO structurally similar to fucosyl-glycans), linking nutrient availability to increased propionate production and immune suppression.^31^ By contrast, in CRC, *M. gnavus* enhances anti-tumor immunity by degrading lysoglycerophospholipids, relieving CD8^+^ T cell inhibition, and protecting against tumor growth.^32^

*M. torques* administration ameliorates DSS-induced autophagy dysregulation in colonic tissues, partially reversing the elevated LC3-II/LC3-I ratio and increasing p62 levels. It induces tight junction proteins expression, reduces pro-inflammatory cytokines TNF-*α* and IL-6, and increases anti-inflammatory IL-10, potentially through bile acid metabolic reprogramming.^151^ *B. thetaiotaomicron* generally plays a beneficial role in IBD but can be harmful depending on environmental context. It alleviates DSS-induced colitis by activating aryl hydrocarbon receptor (AhR) signaling and promoting FOXP3^+^ Treg differentiation,^152^ and reduces pro-inflammatory NF-κB activity in IL-10^-/-^ mice through a pirin-like protein.^153^ However, in high-casein diets, *B. thetaiotaomicron* expansion increases sialidase activity, thins the mucin layer, and worsens DSS colitis.^60^ Loss of sphingolipid production further shifts its effect, inducing goblet cell hyperplasia and macrophage infiltration with increased IL-6 and MCP-1 in colonic lamina propria.^154^

*B. fragilis* is generally protective, particularly via PSA. PSA induces IL-10 and suppresses IL-17, mitigating TNBS- and DSS-induced colitis^91^^,^^155^ as well as AOM/DSS-driven CRC, in a TLR2-dependent manner.^156^^,^^157^ PSA expression is regulated by promoter orientation. The “off” orientation is more common in patients with IBD and colorectal cancer.^158^ Thus, PSA loss or impaired fucosylation diminishes its immunoregulatory effects, promoting colitis.^159^

### 4.2 Extra-intestinal diseases

Beyond gastrointestinal disorders, MD bacteria influence systemic conditions, including autoimmune, metabolic, neurological, and dermatological diseases. Their effects are mediated through microbial metabolites, microbial-associated molecular patterns (MAMPs), and immune signals that circulate beyond the gut to distant organs.

#### 4.2.1 Allergy and autoimmune diseases

Dysbiosis is a hallmark of allergy and autoimmune diseases, where MD bacteria can exert opposing roles. In children with allergy, *M. gnavus* abundance is elevated, while *Bifidobacterium longum, B. dorei*, and *B. vulgatus* are reduced.^129^ *M. gnavus* strains isolated from allergic children display a distinct phenotype compared to those from healthy individuals, characterized by higher pro-inflammatory induction and epithelial adherence, but lower polysaccharide-degrading capacity.^129^ *A. muciniphila* exacerbates food allergy and induces atopic phenotypes under fiber deprivation, despite its known role in controlling inflammation in the presence of dietary fiber.^25^

Infants receiving mixed breast milk-formula feeding who later develop atopic dermatitis show reduced *A. muciniphila* and *M. gnavus*, along with impaired PRR signaling and antigen processing, essential for immune cell activation and differentiation.^133^ In murine dermatitis models, *A. muciniphila* reduces thymic stromal lymphopoietin, suppresses Th2 cytokines, strengthens gut integrity, and alleviates disease progression.^160^ *M. gnavus* may also contribute protectively by inducing IL-10–producing Tregs.^30^

In systemic lupus erythematosus (SLE) models, *A. muciniphila* supplementation restores microbial balance, lowers systemic cytokines, and improves renal function.^161^ In contrast, *M. gnavus* is enriched in lupus nephritis patients, and isolates from these patients disrupt intestinal barrier integrity more strongly than those from healthy donors.^162^ *B. fragilis* supplementation in MRL/lpr mice (an SLE model) alleviates disease by promoting CD1d expression, suppressing CD86 on B cells, and restoring the Th17/Treg balance.^163^

In rheumatoid arthritis, *A. muciniphila* and *M. gnavus* levels increase while *Bacteroides* decreases. These shifts correlate with mucosal barrier erosion and ankle swelling, suggesting that mucolytic activity contributes to inflammatory joint pathology.^118^

#### 4.2.2 Metabolic diseases

*A. muciniphila* abundance is inversely correlated with obesity and type 2 diabetes.^52^^,^^164^ Supplementation improves gut barrier integrity, reduces fat mass gain and inflammation, and alleviates glucose intolerance and insulin resistance.^52^^,^^165^ Clinical trials confirm benefits in individuals with low baseline abundance, including weight loss, improved HbA1c, and increased fat oxidation.^166^ Mechanistically, *A. muciniphila* adapts to HFD environments by producing CO₂, enhancing its ecological fitness and anti-obesity effects.^167^

By contrast, *M. gnavus* is enriched in obesity, where it disrupts bile acid metabolism, correlates with fat mass, and promotes liver injury.^168^ In HFD-fed mice lacking epithelial *Gpr35*, *M. gnavus* expansion drives liver steatosis.^100^ It is also increased in diabetic nephropathy, where it worsens the disease via tight junction disruption, NLRP3 inflammasome activation, and IL-6 induction.^169^

#### 4.2.3 Neurological disorder

MD bacteria are increasingly implicated in central nervous system (CNS) autoimmunity and neurodegeneration.^23^^,^^170-172^ In patients with MS and experimental autoimmune encephalomyelitis (EAE), *A. muciniphila* frequently expands.^119^ *A. muciniphila*-specific IgG is also elevated in cerebrospinal fluid and positively correlates with MS symptom severity and immune cell infiltration.^173^^,^^174^ Depending on microbial community richness and host context, *A. muciniphila* contributes differentially to disease severity.^175^ Its presence can promote Treg differentiation^176^ and suppress Th17 responses, generally exerting protective effects.^177^ However, in a less complex microbiota ecosystem, *A. muciniphila* actively produces *γ*-amino butyric acid, which is associated with neuroinflammation.^175^ It also produces the encephalitogenic P3 peptide, a protein that exhibits molecular mimicry of myelin and contributes to EAE development.^178^

In Alzheimer’s disease (AD), *A. muciniphila* abundance is reduced along with propionate production,^126^ and exogenous administration improves cognition and barrier integrity.^126^^,^^179^^,^^180^ *Bacteroides* spp. and *B. fragilis* metabolites often promote AD pathology by activating microglia and enhancing tau protein accumulation,^181^^,^^182^ although *B. ovatus* has protective effects via lysophosphatidylcholine production and ferroptosis inhibition.^183^

In Parkinson’s disease (PD), *A. muciniphila* exerts opposing roles: when grown in mucin, it promotes *α*-synuclein misfolding and motor deficits,^172^ but under mucin-free conditions, it elevates butyrate, reduces neuroinflammation, and protects dopaminergic neurons.^184^ *M. gnavus* is also implicated in neurological disorders; monocolonization alters sialic acid metabolism and upregulates neuroplasticity-related genes while promoting pathogenic microglia recruitment.^185^

### 4.3 Microbial interactions and pathogen dynamics

MD bacteria play multifaceted roles in shaping intestinal homeostasis through direct and indirect interactions with infectious microbes. These interactions can result in either protection against or promotion of enteric infections, depending on diet, host immune status, and microbial context.^186^ On the protective side, MD bacteria reinforce the intestinal barrier, produce antimicrobial metabolites, and compete with pathogens for mucosal nutrients. Conversely, they may also support pathogen colonization by altering the gut microenvironment in ways that favor microbial invasion.^187^^,^^188^

Interactions between MD bacteria and co-resident microbes strongly influence whether they protect the host or enable infection. *A. muciniphila* shows context-dependent effects during *Citrobacter rodentium* infection. Under fiber-deficient diets, it exacerbates infection by thinning the mucus layer and increasing epithelial permeability, while under fiber-sufficient conditions, it protects against pathogen invasion.^48^ Expansion of *A. muciniphila* and its succinate production, triggered by increased mucosal galactosylation, further enhances *C. rodentium* virulence factors and increases the host susceptibility.^104^

*B. thetaiotaomicron* facilitates *C. rodentium* and enterohemorrhagic *E. coli* infection by promoting succinate production, which pathogens sense through the Cra transcription factor to upregulate virulence genes.^189^ Yet, in co-culture with pathogenic *E. coli*, both *A. muciniphila* and *B. thetaiotaomicron* can reduce IEC pro-inflammatory responses and enhance tight junction protein expression, underscoring their protective potential under defined conditions.^75^

In *Salmonella* Typhimurium infection, *A. muciniphila* exerts a protective role by producing propionic acid, maintaining the mucus layer, secreting antimicrobial peptides, and activating NLRP3 inflammasome pathways.^190^^,^^191^ However, another study reported a contradictory outcome in which *A. muciniphila* exacerbates infection by reducing goblet cells and promoting pro-inflammatory cytokine expression in the cecum and colon,^192^ while also suppressing *B. thetaiotaomicron*. The discrepancy between these findings appears to stem from the culture conditions: *A. muciniphila* grown under mucin-deficient conditions tends to induce higher mucin-degrading activity during infection without the ability to support mucus turnover due to limited propionic acid production.^33^^,^^192^

*Campylobacter jejuni*, though unable to degrade mucin independently, exploits free L-fucose supplied by MD bacteria such as *B. fragilis* and *B. vulgatus* via fucosidase activity. This interdependence enables *C. jejuni* colonization and highlights how MD species can inadvertently provide metabolic support for pathogens.^187^^,^^188^

### 4.4 Determinants of beneficial vs. pathogenic outcomes

The high adaptability of MD bacteria underlies their dualistic roles in both gastrointestinal and systemic diseases. These contrasting outcomes can be traced to several interrelated determinants.^193^ Factors such as nutrient availability, host genetic and immune status, strain-level variation, and microbial interactions collectively dictate whether MD activity contributes to health or disease (Table 3).

**Table 3.** Factors affecting MD bacteria behavior.

MD bacteria	Beneficial role				Detrimental role			
	Determinant	Effect	Experimental approach	Ref	Determinant	Effect	Experimental approach	Ref
*Akkermansia muciniphila*	Mucin-enriched	Propionic acid production	RAW 264.7 cells	[33]	Fiber depletion	Increase MD activity	*C. rodentium* infection, ovalbumin sensitization, peanut sensitization	[25,48]
		Propionic acid production,mucin barrier integrity	*Salmonella* Typhimurium infection	[190,191]	Serine depletion	*A. muciniphila* overgrowth, increase MD activity	DSS-induced colitis	[62]
					Mucin-deficient	Increase pro-inflammatory cytokines	RAW 264.7 cells	[33]
					Galactosylated mucus	Succinate production	*C. rodentium* infection	[104]
*Mediterraneibacter gnavus*	Not excessive sialic acid	Arginine metabolites and Nos2 production	*M. gnavus* mono-colonized mice	[54]	Absence of capsular polysaccharide	TNFα production	BMDCs	[88]
	Fucosyl-glycan	Propionate	IL10-/- mice,	[31,33]				
*Bacteroides thetaiotaomicron*						Succinate production	*C. rodentium* infection*, Clostridium difficle* infection	[189,194]
					High casein	Increase sialidase activity	DSS-induced mice	[60]
					Lacking sphingolipid production capability	Goblet cell hyperplasia, macrophage infiltration	Mono-colonized germ-free mice	[154]
*Bacteroides fragilis*	PSA	IL-10 production, immunoregulatory properties	Splenic DCs, PBMC derived plasmacytoid DC, *H. hepaticus*-induced colitis	[90,91]				
*Bifidobacterium bifidum*	Sialidase activity	Increase barrier integrity	Caco-2 monolayer	[195]				

#### 4.4.1 Nutrient availability and diet

Dietary composition is one of the strongest modulators of MD bacterial behavior. The absence of dietary fiber drives excessive mucin degradation, leading to lethal colitis phenotypes,^71^ aggravation of food allergy,^25^ and increased susceptibility to pathogen infection.^48^ These effects are not observed under fiber-rich diets, underscoring how nutrient availability shapes MD activity and disease outcomes.^71^ Amino acid composition is equally critical. Because MD bacteria have distinct amino acid requirements, the presence of certain amino acids can accelerate growth and beneficial functions, while their absence may shift the balance toward competition with commensals and promote pathogenic behavior.^62^

Accessible mucus glycans also modulate the immunogenic and metabolic properties of MD bacteria, influencing whether they act protectively or pathologically. In the presence of accessible sialic acid, *M. gnavus* increases its adaptability in the inflamed gut^13^ and reduces production of beneficial metabolites important for maintaining intestinal barrier integrity.^54^ Conversely, when fucose is available, *M. gnavus* promotes propionic acid-mediated immunomodulation.^31^^,^^33^ Furthermore, excessive galactosylation induces succinate production by *A. muciniphila*, enhancing its ability to exacerbate pathogen infection.^104^

#### 4.4.2 Strain-level variation

Even within a single species, MD bacteria exhibit substantial genotypic and phenotypic diversity that drives their context-dependent effects.^146^ Differences in GH and CBM repertoires determine adaptability, mucin utilization, and metabolite production.^148^^,^^196^ Nan-dependent mucin-degrading capacity of *M. gnavus* is highly strain-specific: five strains (including the type strain ATCC 29149) carry the full *nan* operon with an ABC transporter, five strains lack *nan* genes entirely, and two strains encode only partial *nan* modules (*nanA*/*nanE*/*nanK*).^197^ These differences indicate that the ability to utilize 2,7-anhydro-Neu5Ac or Neu5Ac varies markedly across strains. Immunomodulatory properties are also strain dependent. For instance, *M. gnavus* strains produce exopolysaccharides and capsular polysaccharides, eliciting markedly different host immune responses.^87^^,^^88^ These strain-level features help explain why MD species can be either protective or pathogenic depending on the context.

*Bifidobacterium bifidum* exhibits substantial strain-level differences in its interaction with the intestinal mucus layer and epithelium.^195^ Probiotic strains W23 and W28 possess high sialidase activity, selectively modify the extracellular domain of MUC13, and adhere more strongly to the mucus layer than the type strain DSM 20456. Despite their robust mucin-degrading capacity, W23 and W28 enhance epithelial barrier integrity under inflammatory conditions, whereas DSM 20456 does not. These findings indicate that modulation of the glycocalyx and epithelial barrier function is highly strain dependent rather than species dependent.

Within the *Bacteroides* genus, *B. ovatus* and *B. xylanisolvens* show pronounced strain-level variation in mucin glycan degradation.^139^ Pangenome analyses reveal a highly heterogeneous distribution of polysaccharide metabolism genes, shaped partly by interspecies gene transfer and driving substantial functional divergence. Transcriptomic data further suggest that some lineages have lost mucin-utilization capacity, retaining only residual or inactive pathways, whereas other strains maintain a complete mucin-degradation system.

Overall, these results underscore that strain-dependent functional heterogeneity is a critical determinant of both probiotic potential and mucin-degrading capabilities.

#### Metabolite production

MD bacteria produce diverse metabolites that act as key communication signals with the host. SCFAs, particularly propionate, exemplify their health-promoting functions, as shown in *A. muciniphila* growing on mucin and *M. gnavus* utilizing fucose.^31^^,^^33^^,^^198^ Conversely, other byproducts can promote pathology. Branched-chain fatty acids, derived from protein fermentation, and succinate, produced under vitamin B_12_ limitation or enhanced mucosal galactosylation, have been linked to increased inflammation and greater susceptibility to pathogen infection.^33^^,^^40^^,^^104^^,^^198^ Thus, the metabolic outputs of MD bacteria are central determinants of their beneficial versus pathogenic outcomes. Beyond these fermentative products, MD bacteria also produce a variety of amino acid- and lipid-derived metabolites that function as critical signaling molecules. These include indoles,^33^ ornithine lipids,^80^ and sphingolipids,^154^ which collectively modulate host immune responses, reinforce intestinal barrier integrity, and regulate host metabolic pathways. Among MD species, *M. gnavus* is particularly notable for its metabolite-driven links to IBD, obesity, and diabetes mellitus. It produces immunomodulatory glycopolysaccharides^29^ as well as other bioactive molecules, including ursodeoxycholic acid, hydrogen peroxide, butyrate, and glycine ursodeoxycholic acid. These metabolites influence cytokine production, epithelial barrier function, and systemic metabolic regulation.^199^

Taken together, these determinants highlight why MD bacteria act as “double-edged swords.” Their activities can reinforce mucosal integrity, immune tolerance, and systemic health, yet under altered nutrient, genetic, or microbial contexts, they may instead exacerbate inflammation, pathogen invasion, or tumor progression. Understanding the interplay of diet, host immunity, strain variation, and microbial ecology is essential for leveraging MD bacteria as therapeutic targets or next-generation probiotics.

## 5 Conclusions and future perspectives

Although MD bacteria represent only a small fraction of the gut microbiota, they are highly responsive to alterations in the intestinal ecosystem. Through mucin utilization and their associated metabolic activities, MD bacteria exert a disproportionate influence on both microbe–microbe and host–microbe interactions. Importantly, their roles cannot be reduced to a simple dichotomy of “beneficial” versus “detrimental.” Instead, MD bacteria display highly context-dependent effects determined by key factors including strain-specific genetic variation, nutrient availability, amino acid preferences, host genetics and immunity, and the overall microbial ecosystem. Through these interactions, MD bacteria can either reinforce barrier integrity and immune tolerance or promote dysbiosis, inflammation, and pathogen invasion.

The context-dependency is reflected in their mucolytic enzymes repertoires, MAMPs, and metabolic products. Therefore, rather than relying solely on abundance, functional features such as enzyme expression, glycan-targeting behavior, and metabolite production may serve as more accurate biomarkers of disease risk and mechanistic involvement.

This duality also presents an opportunity: by understanding the ecological and molecular determinants that drive MD bacteria toward beneficial functions, it may be possible to program their activity to sustain gut homeostasis. Future studies should investigate additional factors involved in MD bacterial adaptability to better define their role in intestinal ecosystem orchestration. Potential strategies include the selection of specific probiotic strains, dietary interventions tailored to nutrient requirements, or microbiome-targeted therapies that encourage cross-feeding while suppress pathogenic pathways. Harnessing strain-specific metabolic and immunomodulatory properties has the potential to support the development of next-generation probiotics and live biotherapeutic products that reinforce epithelial barrier function, fine-tune mucosal immunity, and promote durable anti-inflammatory states. By prioritizing strains with favorable mucin-modulating profiles and pairing them with nutrient environments that support balanced mucus turnover, these interventions could convert context-dependent residents into reliably beneficial partners in maintaining gut and systemic health.

While well-studied model organisms such as *A. muciniphila*, *M. gnavus*, and *Bacteroides thetaiotaomicron* have advanced our understanding, the functional roles and regulatory determinants of many other MD bacteria remain poorly characterized. Expanding research into these lesser-known taxa—through strain isolation, multi-omics analysis, and mechanistic validation—will be critical for defining their contributions to both health and disease.

In sum, MD bacteria should be viewed as adaptive modulators of the gut ecosystem whose effects depend on strain, diet, host, and microbial context. Harnessing this plasticity offers a promising avenue for next-generation microbiome-based interventions aimed at preventing or treating gastrointestinal and systemic diseases.

## Data Availability

Data sharing is not applicable to this article as no new data were created or analyzed in this study.
